# Supplementary material for: The Global Health Security Index is not predictive of coronavirus pandemic responses among Organization for Economic Cooperation and Development countries
Source: PLoS One. 2020 Oct 7;15(10):e0239398. doi: 10.1371/journal.pone.0239398 (PMC7540886; doi:10.1371/journal.pone.0239398)
Supplement: Attachment — Submitted filename: Response to Reviewers_OECD_8.31.2020.doc [file pone.0239398.s001.doc]

JOHNS HOPKINS

# UNIVERSITY

## Division of Infectious Diseases

### School of Medicine

1550 Orleans Street, Room 110

Baltimore, Maryland 21287

(410) 502-8233 / FAX (410) 614-8173

E‑mail: [petros@jhmi.edu](mailto:wbishai1@jhmi.edu)

### Center for Tuberculosis Research

Petros C. Karakousis, M.D.

August 31, 2020

Itamar Ashkenazi

Academic Editor

PLOS ONE

Re: PONE-D-20-22950

Dear Dr. Ashkenazi,

We appreciate the opportunity to revise our manuscript entitled, “The Global Health Security Index is not Predictive of Coronavirus Pandemic Responses among Organization for Economic Cooperation and Development (OECD) Countries”, which we would like to submit for consideration for publication in PLoS ONE.

We thank the Reviewer and the Editor for their thorough review of our manuscript and for the constructive feedback. We have included point-by-point responses to each of the Reviewer’s comments and made the appropriate revisions in the manuscript, which we believe is significantly improved as a consequence.

Sincerely,

Petros C. Karakousis, M.D.

Professor of Medicine

Reviewer Recommendation and Comments for Manuscript Number PONE-D-20-22950

Reviewer #1:

Please note that the NTI / GH Index authors have not been in direct contact with key people responsible for Emergency Preparedness in their country in order to make a real assessment.... they just summarized publications that were published.... that a significant fault that causes misleading conclusions about scoring the level of preparedness, effectiveness of preparedness etc.......

Response: We thank the Reviewer for the insightful comment. We agree that the one of the limitations of the GHS Index is the failure to engage key persons responsible for emergency preparedness in their respective countries and other key stakeholders. This point has been discussed in the revised manuscript (lines 187-189).
